# Deep Ganglionic Intracerebral Hemorrhage Due to Cavernous Malformation Mimicking Hypertensive Hemorrhage: A Report of Two Cases

**DOI:** 10.7759/cureus.36448

**Published:** 2023-03-21

**Authors:** Hiroki Kobayashi, Takeshi Ogura, Kazuma Kowata, Mayu Nakajima, Shigehiro Ohmori, Hiroki Kurita

**Affiliations:** 1 Department of Neurosurgery, International Medical Center, Saitama Medical University, Saitama, JPN; 2 Department of Neurosurgery, Kurosawa Hospital, Gunma, JPN

**Keywords:** intracerebral hemorrhage, hypertensive intracerebral hemorrhage, cerebral cavernoma, deep ganglionic cavernous malformation, cavernous malformation

## Abstract

Cavernous malformation (CM) is a type of vascular malformation that is an important cause of intracerebral hemorrhage. However, because CM is a low-flow vascular malformation, the occurrence of major hemorrhage is rare. We present two patients with deep ganglionic intracerebral hemorrhage that caused a significant mass effect, mimicking hypertensive hemorrhage. In both cases, we performed evacuation of the hematoma as a lifesaving treatment and made a pathological diagnosis of CM. In conclusion, preoperative diagnosis of CM using any kind of radiological evaluation is difficult, especially in patients with major hemorrhage. The possibility of CM should be remembered in cases with deep ganglionic intracerebral hemorrhage.

## Introduction

Cavernous malformation (CM) is a type of vascular malformation that is an important cause of intracerebral hemorrhage (ICH) and accounts for 5-15% of all intracranial vascular malformations [1-3]. They are present in 0.5% of the population and usually present with seizures, focal neurological deficits, stroke, and headaches [4,5]. The annual rate of hemorrhages of CM ranges from 0.7% to 1% [6]. CM is an angiographically occult and low-flow vascular malformation, and although it often leads to the oozing of blood from the lesion, major hemorrhage is rare [7]. Hence, CMs are usually not considered in the differential diagnosis of major hemorrhage lesions, especially when a significant mass effect is present. Although there might be cases of major hemorrhage with a significant mass effect caused by CM and whose radiological findings might occasionally mimic hypertensive ICH, only a few such cases have been reported. Here, we report two cases of CM in the basal ganglia that caused major hemorrhage with a significant mass effect, mimicking hypertensive hemorrhage.

## Case presentation

Case one

**Figure 1:**
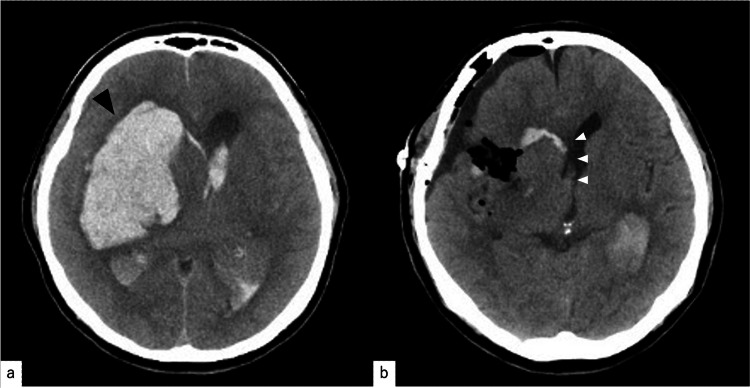
(a) Initial computed tomography (CT) in Case one: hemorrhage (measuring 8.5 × 5.3 × 4 cm in diameter with a volume of 90.1 mL) in the right basal ganglia region (black arrow). (b) Postoperative CT: almost complete removal of the lesion with a significant reduction in the midline shift (white arrow).

**Figure 2:**
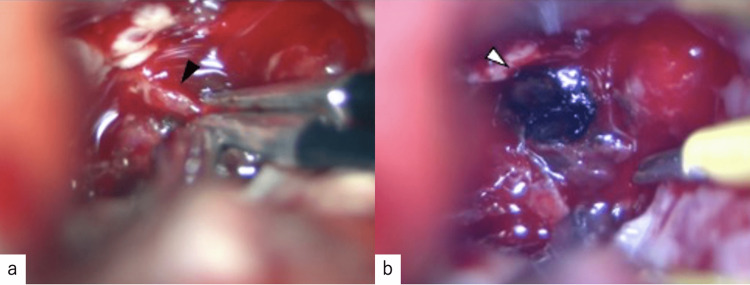
(a) Small feeders (black arrow) to the cavernous malformation were coagulated using bipolar forceps. (b) A hemorrhagic pouch-like structure (white arrow) was visualized inside the hematoma.

**Figure 3:**
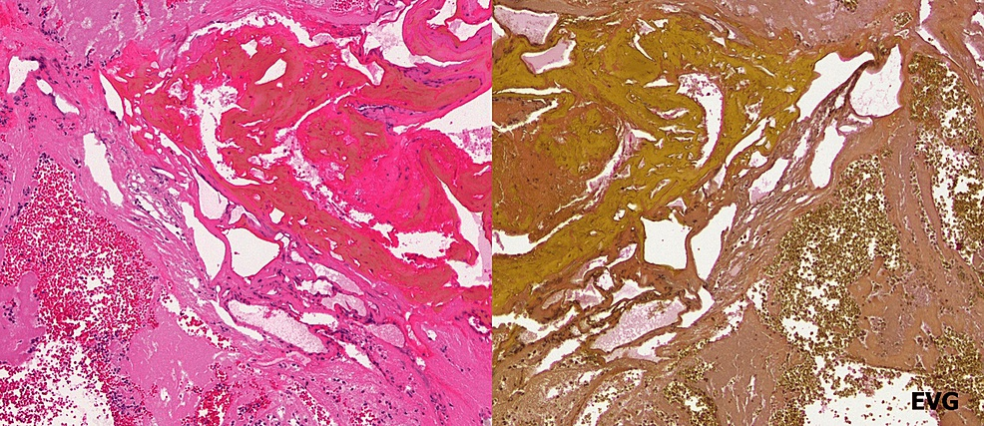
Microscopic images showing the presence of a degenerative vascular structure that lacked smooth muscle structures in the vessel wall, with abnormal dilation of blood vessels (hematoxylin and eosin staining and Elastica van Gieson staining, magnification ×10).

A 48-year-old man was admitted to our emergency department due to left hemiplegia and progressive consciousness disturbance. He had no past medical history. On admission, his Glasgow Coma Scale score was 4 (E1V1M2) and he was in a deep coma. His blood pressure was 198/124 mmHg, and his heart rate was 64 beats per minute. Physical examination was significant for paralysis of the left upper and lower limbs, his right and left pupils were 5.0/3.0 mm in size, and the pupillary light reflex was altered. Head non-contrast computed tomography (CT) revealed intracerebral hematoma in the region of the right basal ganglia (measuring 8.5 × 5.3 × 4 cm in diameter with a volume of 90.1 mL), intraventricular hemorrhage, and a midline shift to the left (Figure 1). To address impending brain herniation, we decided to perform evacuation of the hematoma as a lifesaving treatment. Intraoperatively, while performing evacuation of the hematoma around the basal ganglia, we noticed a hemorrhagic pouch-like structure with persistent oozing of blood. After removing the structure and the feeding arteries surrounding it by bipolar coagulation, complete hemostasis in the hematoma cavity was achieved (Figure 2). Histological examination showed a degenerative vascular structure in the hematoma with no smooth muscle structure in the vessel wall, but with abnormal dilation of blood vessels, compatible with a CM (Figure 3). Postoperatively, the left hemiplegia and consciousness disturbance (Glasgow Coma Scale score of 6: E2VTM4) persisted. The patient underwent a tracheostomy on day 10 of hospitalization and was transferred to another hospital for rehabilitation on day 92 of hospitalization.

Case two

**Figure 4:**
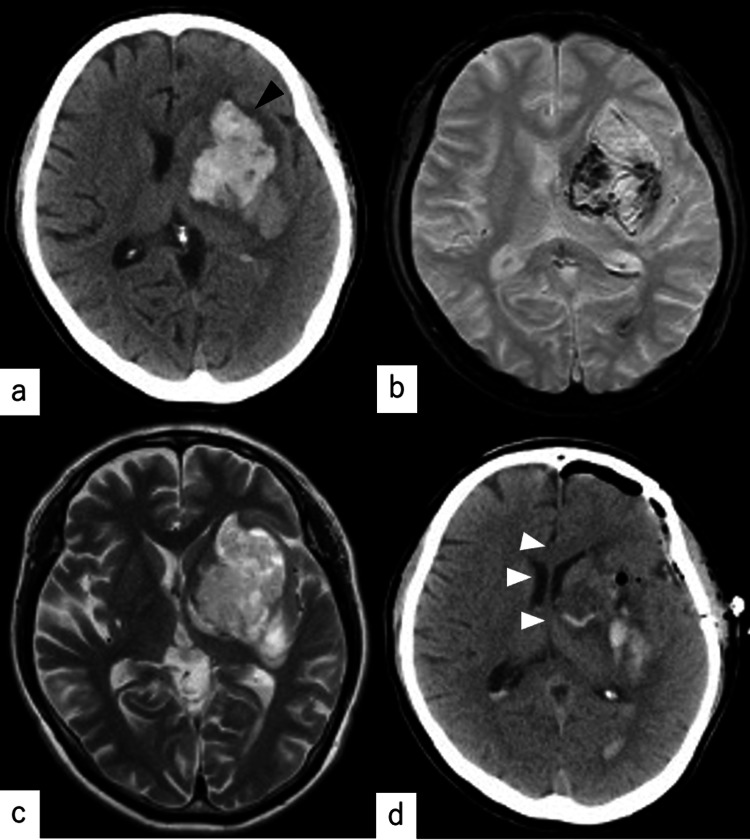
(a) Initial computed tomography (CT) in case 2: hemorrhage in the left basal ganglia (measuring 6.7 × 4.4 × 3 cm in diameter with a volume of 44.2 mL) (black arrow). (b-c) Magnetic resonance imaging (MRI): conventional T2-weighted imaging and T2-weighted imaging with a gradient-echo MRI sequence did not reveal the cavernous malformation. (d) Postoperative CT: the lesion was almost completely removed and there was a reduction in the midline shift (white arrow).

**Figure 5:**
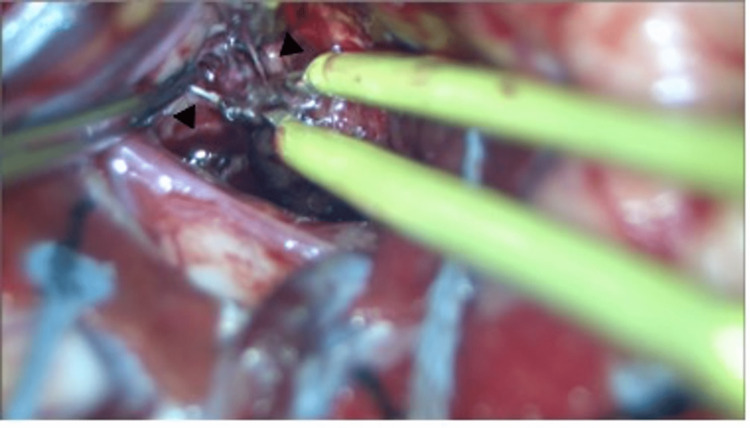
The small vessels of the cavernous malformation (black arrow) were coagulated using bipolar forceps.

**Figure 6:**
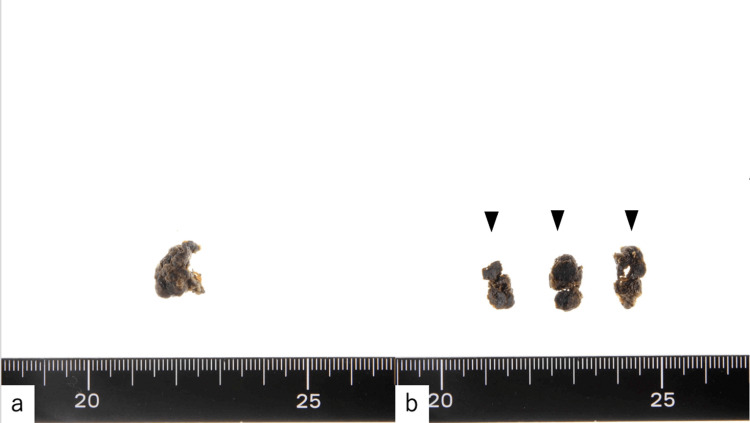
(a) Macroscopic evaluation of the small pouch-like lesion. (b) Cross-section surfaces of the lesion (black arrows).

**Figure 7:**
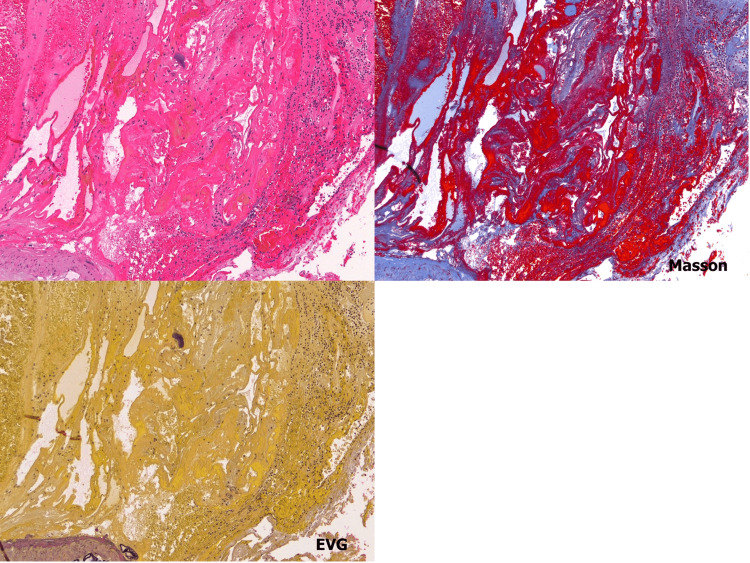
A large vessel with a thin membranous structure suggestive of fragile collagen fiber tissue was identified. There was no evidence of elastic fiber intervention on Elastica van Gieson (EVG) staining, and there was thin membranous collagen fiber tissue inside the hematoma and cavernous-like structure on Masson trichrome staining (hematoxylin and eosin stain, EVG stain, and Masson trichrome stain, magnification ×10).

A 59-year-old man was admitted to our emergency department due to consciousness disturbance, right hemiplegia, and left conjugate eye deviation. His medical history was significant only for urolithiasis. On admission, his Glasgow Coma Scale score was 9 (E2V3M4), his blood pressure was 144/69 mmHg, and his heart rate was 89 beats per minute. Physical examination was significant for paralysis of the right upper and lower limbs, his pupils were 2.0/2.0 mm in size with intact pupillary light reflex, and he had aphasia and left conjugate eye deviation. Head non-contrast CT revealed intracerebral hematoma in the left basal ganglia (measuring 6.7 × 4.4 × 3 cm in diameter with a volume of 44.2 mL) and midline shift to the right. Magnetic resonance (MR) angiography did not show any vascular anomaly and there was no evidence of other hemorrhagic lesions on T2-weighted MR imaging with a gradient echo sequence (Figure 4). To prevent brain herniation, the patient underwent an emergency craniotomy for evacuation of the hematoma. Intraoperatively, a cyst-like structure with small feeding arteries was identified inside the hematoma cavity (Figure 5). Hemostasis was achieved with coagulation of these surrounding arteries and the entire structure was removed as one structure. On macroscopic evaluation, the lesion had a pouch-like structure (Figure 6). On histological examination, although a large vessel with a thin membranous structure of collagen fiber tissue was identified, the collagen fiber tissue was very fragile and it was difficult to differentiate it from a fibrin clot with hematoxylin-eosin staining. Elastica van Gieson (EVG) staining and Masson trichrome staining were also performed. EVG staining showed that there was no evidence of elastic fiber intervention, and Masson trichrome staining showed thin membranous collagen fiber tissue inside the hematoma, along with abnormal dilation of blood vessels (Figure 7). These findings confirmed a diagnosis of CM. Postoperatively, the right hemiplegia, aphasia, and consciousness disturbance (Glasgow Coma Scale score of 12; E4V3M5) persisted and he was transferred to another hospital for rehabilitation on day 30 of hospitalization.

## Discussion

CM is the most common vascular malformation, which can cause severe neurological symptoms such as hemorrhagic stroke (30-40%), seizures (40-70%), headaches (10-30%), and focal neurological symptoms (35-50%) [8]. Reportedly, the location of CM is lobar in 66%, brainstem in 18%, deep supratentorial in 8%, and cerebellar in 8% of cases [9]. Although deep-seated CMs, including in the basal ganglia, are relatively rare lesions, they have been reported to increase the risk of hemorrhage and can lead to considerable neurological impairment because of their vital location [10-13].

CMs consist of a cluster of enlarged capillary-like channels with a single layer of endothelium and without intervening brain parenchyma [14]. Macroscopically, they appear as mulberry-like or hematoma-like lesions, although the findings vary from case to case. Because CMs are low-pressure lesions, they are angiographically occult, and their diagnosis is more difficult than that of other vascular diseases. On CT, CMs might appear as a well-demarcated mass with either homogeneous or heterogeneous high-density-related thrombosis, calcification, increased blood volume, and hemosiderin deposition [15]. Angiography is only able to detect the presence of abnormal venous drainage associated with CM and not the CM itself. Hence, other imaging techniques are needed for an accurate diagnosis.

MRI is a useful technique for the diagnosis of CM [16,17]. On MRI, CM is characterized by the presence of microhemorrhage surrounding the vascular malformation. Hemoglobin-degeneration products of methemoglobin, hemosiderin, and ferritin allow for its detection on MRI [18,19]. The MRI features of CM also include a reticulated pattern of mixed hyper- and hypointensity on conventional T1- and T2-weighted imaging, or a characteristic hypointense rim that is best appreciated on T2-weighted imaging with a gradient-echo sequence [16,20]. Susceptibility-weighted imaging is also very useful for detecting CMs because it accurately recognizes deoxyhemoglobin and hemosiderin [21].

In our cases, the major ICH was located in the region of the basal ganglia, and their radiological findings on CT and MRI mimicked hypertensive hemorrhage. CMs are low-flow vascular malformations that are usually not considered a cause of major ICH. To our knowledge, there are no previous reports of cases of life-threatening hematoma in the deep basal ganglia due to CM. However, it is reported that a non-negligible number of ICH cases are due to causes other than hypertension [22]. The frequency of vascular malformations in ICH reportedly varies between 4% and 8% [23]. Due to their tiny and fragile structure and the effect of coagulation during surgery, it is possible that many cases, as in this report, might have been overlooked.

Therefore, we must always consider vascular malformations, including CMs, in the differential diagnosis of patients with ICH regardless of the volume of the hematoma. When intraoperative findings suggest the presence of CM, complete surgical removal should be the goal of treatment to prevent postoperative hemorrhage because residual CMs have been reported to increase the risk of re-hemorrhage [24-26].

## Conclusions

We report two cases with deep ganglionic ICH that caused a significant mass effect, mimicking hypertensive hemorrhage. Major ICH is a critical medical emergency, which often requires surgical intervention despite minimal information about the etiology. Although CM as the cause of major ICH in the region of basal ganglia is rare and difficult to diagnose preoperatively based on radiological studies, we must keep the various etiological possibilities other than hypertension in mind in patients with ICH. Careful exploration of the hematoma cavity to identify vascular malformations is essential for preventing postoperative hemorrhage.
